# Detection of Ki-ras mutations in tissue and plasma samples of patients with pancreatic cancer using PNA-mediated PCR clamping and hybridisation probes

**DOI:** 10.1038/sj.bjc.6602319

**Published:** 2005-01-11

**Authors:** J Däbritz, J Hänfler, R Preston, J Stieler, H Oettle

**Affiliations:** 1 Charité – Universitätsmedizin Berlin, Campus Virchow-Klinikum, Medizinische Klinik und Poliklinik m.S. Hämatologie und Onkologie, Augustenburger Platz 1, 13353 Berlin, Germany

**Keywords:** Ki-ras, real-time PCR, PNA, pancreatic cancer

## Abstract

In the present study, we combined the PCR-clamping approach with melting curve analysis using mutant specific hybridisation probes and wild-type specific peptide nucleic acids (PNAs) to determine the genotypes of the most frequent point mutation in codon 12 of the proto-oncogene Ki-ras in tissue and plasma samples of patients with pancreatic cancer. The sensitivity of our assay was 1–5 × 10^−5^. The melting curve analysis of tissue samples of four patients revealed two valine mutations, one none-valine mutation and one wild-type sequence. Ki-ras alterations were found in 28% of DNAs (18 out of 64) of nonrelated plasma samples of 10 patients with ductal adenocarcinoma of the pancreas. The valine mutation was the predominantly detected gene alteration (83%). Out of ten patients investigated, four patients (40%) became positive during clinical observation with respect to Ki-ras mutation. All four patients exhibited progressive disease and high levels of tumour marker CA 19-9. In conclusion, the one-step procedure discribed may be a useful clinical tool for analysing Ki-ras point mutations in tissue and plasmas samples. In addition, this method can be adapted for simultanous detection of multiple mutations and quantitation.

Pancreatic cancer remains a major cause of death in western populations. Despite many efforts, little is known about its aetiology (Malats, 2001). Several risk factors have been implicated: male gender, black race, cigarette smoking, diabetes mellitus and meat and fat consumption (Sakorafas *et al*, 2000; Sternheim *et al*, 2000; Simon and Printz, 2001).

Ductal adenocarcinoma of the pancreas is a highly aggressive tumour with an early tendency of spreading to other organs (Hermanek, 1998). Pancreatic cancer has a poor prognosis. The median survival is less than 6 months. The overall 5-year survival of 3% associated with pancreatic cancer is largely a result of diagnosis late in the course of the disease. Surgical resection remains the only chance for cure in patients with pancreatic cancer, but only 15–20% of patients have lesions that are resectable at the time of diagnosis. Current postresection 5-year survival rates are approximately 20% (Wong *et al*, 2001). Conventional tumour markers, such as CA 19-9, have a high diagnostic sensitivity (80–90%), but lack a specifity (50–70%) and are therefore not suited for detecting early tumours (Berndt *et al*, 1998; Lamerz, 1999; Wenger *et al*, 1999). More effective screening techniques are urgently needed to improve the poor prognosis of the disease (Mizumoto and Tanaka, 2002).

Pancreatic cancer is among the best-described genetic diseases (Hilgers and Kern, 1999). The development and growth of pancreatic adenocarcinoma involves oncogene activation, loss of tumour suppressor gene function and overexpression of receptor-ligand systems (Goggins *et al*, 1999; Efthimiou *et al*, 2001; Shi *et al*, 2001). The Ki-ras oncogene is one of the three members of the human ras gene family that code for the highly related 21-kDa proteins with guanosine triphosphatase (GTPase) activity. Naturally occurring mutations in the Ki-ras gene have been localised to codon 12 (most frequently), 13 and 61. Owing to an associated inappropriate stimulation signal, the mutant ras transmits a constitutive growth signal to the nucleus (Gansauge *et al*, 1996; Olson and Marais, 2000). The incidence of ras gene mutations varies among different kinds of tumours, but was found mutated predominantly in pancreatic adenocarcinomas with a frequency of 80–90% (Sirivatanauksorn *et al*, 1998; Ghaneh *et al*, 2002).

Gene alterations of the ras type can be used as molecular markers to screen for the presence of neoplastic cells in different clinical specimens. Enriched PCR methods are able to discriminate mutant from wild-type alleles and also provide a high degree of sensitivity to detect mutant alleles in a large excess of normal DNA. Two PCR-based techniques have been most commonly used: ASA-PCR (allele-specific amplification), which is restricted to the analysis of individual codon-specific mutations and requires several allele-specific oligonucleotides, and the time-consuming RFLP-PCR (restriction fragment length polymorphism), which bears an increasing risk of *Taq* polymerase-born infidelity (Weber, 1990; Kahn *et al*, 1991; Takeda *et al*, 1993; Jacobson and Mills, 1994; Mora *et al*, 1998).

More recently, the mutation-sensitive hybridisation profile of peptide nucleic acids (PNAs) has been exploited to design novel protocols (PCR-clamping). PNAs are none extendable oligonucleotides where the ribose-phosphate backbone is completely replaced by (2-aminoethyl)-glycine units linked by amide bonds. These synthetic oligomers bind with higher thermal stability to complementary nucleic acids than DNA and RNA. On the other hand, the PNA/DNA duplex is significantly destabilised in the case of single-base mismatch (Ørum *et al*, 1993; Ratilainen *et al*, 1998). In PNA-mediated PCR clamping, PNA oligomers suppress amplification of the wild-type sequence confined by a pair DNA oligonucleotide primers (competetive clamping) because PNA oligomers are no substrate for DNA polymerases. In the case of a single-base mismatch, the DNA/PNA duplex is destabilised, which allows strand elongation from bound DNA oligomer to proceed, resulting in the detection of PCR fragments, most of which harbour the variant allele, and the suppression of wild-type genomic sequences during amplification, respectively. This approach has been described for the detection of Ki-ras and p53 point mutations (Thiede *et al*, 1996; Behn *et al*, 2000).

Analysis of PCR products via mutation-specific hybridisation probes guarantees the most specific measurement of amplified target sequences. Hybridisation probes consists of two terminally fluorescent-labelled oligomers. Only after binding to the target sequence in close proximity, fluorescence resonance energy transfer (FRET) occur (Lay and Wittwer, 1997) and the real-time fluorescence can be monitored. Hybridisation probes are furthermore suitable for experiments in which the amplification products formed are subsequently quantified using the evaluation software. The combination of detection by internal hybridisation probes and subsequent melting curve analysis expands the spectrum to include mutation analysis by rapid genotyping. One mismatch only, due to a single point mutation, between the internal hybridisation probes and their target sequences, can drastically change the melting temperature of the bound probe (Lyon, 2001). In a recent study, the PNA-mediated PCR clamping method was combined with on-line detection for c-kit proto-oncogene point mutations (Sotlar *et al*, 2003).

In the present study, we combined the PCR-clamping approach using a wild-type PNA (17-mer) with real-time PCR using mutant-specific hybprobes in single closed LightCycler capillaries. As a preliminary study, plasma and tissue samples of patients with pancreatic cancer were analysed with respect to point mutations in codon 12 of the Ki-ras proto-oncogene by melting point analysis.

## MATERIAL AND METHODS

### Patients

A total of 64 sera from 10 patients (seven male, three female) with pancreatic carcinoma (resectable and nonresectable) were analysed for Ki-ras mutation. In addition, four tissue samples from nonrelated pancreatic tumours were analysed for Ki-ras mutation.

### Cell lines

Cells of the cell line SW 480 (colon carcinoma) harbouring a homozygous Ki-ras codon 12 mutation were cultured in RPMI 1640 medium (50 ml culture flask) supplemented with 10% (v v^−1^) heat-inactivated fetal calf serum and 100 *μ*g ml^−1^ streptomycin (humidified atmosphere, 5% CO_2_, 37°C). Adherent cells were trypsinised and washed once with PBS solution (phosphate-buffered saline).

### DNA extraction from the plasma

Blood samples (9 ml) were withdrawn from a peripheral vein and placed in tubes containing EDTA. The collected samples were centrifuged at 1500 r.p.m. for 10 min (Minifuge RF, Heraeus, Hanau, Germany). Plasma was stored at −20°C until further use. DNA was extracted from plasma with QIAamp spin columns (QIAamp DNA Mini Kit, Qiagen, Hilden, Germany) according to the manufacturer's instruction. Incubation with proteinase K was performed for 10 min at 68°C. Extracted DNA from 200 *μ*l of plasma was eluted with 50 *μ*l and stored at −20°C.

### Isolation of DNA from paraffin-embedded tissue

Tumour tissues were obtained at surgery and fixed with paraffin. DNA was extracted from tissue with QIAamp spin columns (QIAamp DNA Mini Kit) according to the manufacturer's instruction. In brief, not more than 25 mg of paraffin-embedded tissue was placed into a 2 ml microcentrifuge tube. Removal of paraffin was ensured by adding and mixing 1200 *μ*l xylene. After centrifugation at full speed for 5 min at room temperature, the supernatant was carefully removed and 1200 *μ*l of ethanol (96–100%) was added to the pellet. The mixture was centrifuged at full speed for 5 min at room temperature; afterwards, the ethanol was carefully removed. To remove residual xylene completely, the procedure was repeated. The open microcentrifuge tube was incubated at 37°C for 10–15 min until the ethanol had evaporated and the DNA was extracted was with QIAamp spin columns (QIAamp DNA Mini Kit, Qiagen, Hilden, Germany) according to the manufacturer's instruction. Lysis in the presence of proteinase K was overnight at 56°C. AL (200 *μ*l) buffer was added and mixed. DNA was eluted with 100 *μ*l of AE buffer and stored at −20°C.

### Isolation of DNA from SW480 cells

Cells were resuspended in 200 *μ*l of PBS solution. After adding 20 *μ*l proteinase K and 200 *μ*l AL buffer, the mixture was incubated at 56°C for 10 min. DNA extraction was continued as with tumour tissue (see above).

### Real-time PCR and melting curve analysis

Real-time PCR was performed in a final volume of 20 *μ*l containing 10 mM TRIS-HCl pH 8.3, 50 mM KCl, 3.75 mM MgCl_2_, 125 *μ*M of each desoxynucleotide triphospate (Invitrogen, Carlsbad, USA), 1 *μ*M of primer Ki-ras F (5′-AAG GCC TGC TGA AAA TGA CTG-3′) and 1 *μ*M Ki-ras R (5′-GGT CCT GCA CCA GTA ATA TGC A-3′), 0.3 *μ*M hybridisation probe Ki-ras FL (donor) labelled on its 3′-end with fluorescein (5′-CGT CCA CAA AAT GAT TCT GAA TTA GCT GTA TCG TCA AGG CAC T-F-3′) and 0.3 *μ*M Ki-ras LC (acceptor) labelled on its 5′-end with the fluorescence dye LightCycler™-Red 640 (5′-LC Red640-TTG CCT ACG CCA ACA GCT CCA A-P-3′), 2.5 *μ*M PNA 17 mer (5′-CCT ACG CCA CCA GCT CC-3′), Protein Amplifly and 1.25 U of Platinum *Taq* DNA polymerase (Invitrogen). After an initial denaturation step at 95°C for 3 min, 45 cycles were performed with each cycle consisting of: denaturation at 95°C for 10 s, PNA annealing at 76°C for 7 s, annealing of the primers and probes at 60°C for 15 s and elongation at 72°C for 20 s. PCRs were carried out on the LightCycler Instrument (Roche Diagnostics, Mannheim, Germany).

Melting curve analysis was performed at steadily increasing temperature from 40 to 85°C with a transition rate of 0.3°C s^−1^. Fluorescence data obtained were analysed using the LightCycler software (software version 3.5, Roche Diagnostics).

### Enriched *Bst*NI RFLP/PCR

Enriched *Bst*NI RFLP/PCR was confirmed as described elsewhere (Mora *et al*, 1998). Digestion of the amplified products with the restriction enzyme *Bst*NI (New England BioLabs, Beverly, USA) was performed according to the manufacturer's instruction. After two rounds of PCR (heminested PCR) and digestions of the amplified DNA, all PCR products were electrophoresed in a 3% agarose gel (low melting point agarose) and the mutant allele (143-bp band) and the wild-type allele (114-bp band) was visualised by ethidium bromide staining (0.5 mg ml^−1^). All synthetic oligonucleotides used were purchased from TIB Molbiol (Berlin, Germany).

## RESULTS

### Determination of interassay variability and optimisation of PNA concentration

**Figure 1:**
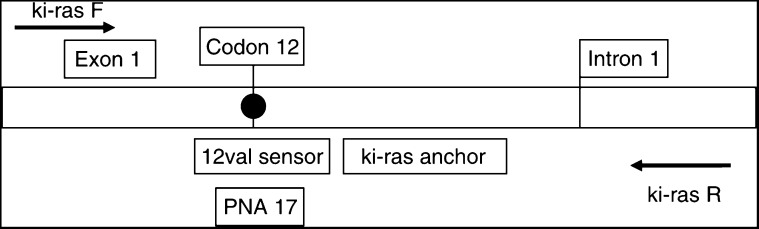
Relative positions and orientations of PCR primers (ki-ras F; ki-ras R), hybridisation probes (12val sensor; ki-ras anchor) and the wild-type peptide nucleic acid (PNA-17) for detection of codon 12 mutation of the Ki-ras proto-onkogene (Genbank accession no. K01519; nucleotid positions 1–164).

For detection of the hot spot point mutation of the Ki-ras proto-oncogene at codon 12, we used PNA-mediated PCR-clamping and real-time PCR with mutant-specific hybridisation probes. The relative positions of primers, hybridisation probes and PNA are shown in Figure 1 (Genbank accession no. K01519; nucleotid positions 1–164). For PNA and hybprobes, the antisense strand was chosen due to the lower purine content to reduce mismatches. After optimising the PCR conditions, the interassay variability of the melting temperatures was determined. For wild-type and valine-mutated DNA, the variability was 68.0°C±0.8 (*n*=15) and 71.2°C±0.8 (*n*=15), respectively, and therefore allows precise discrimination of wild-type and mutant DNA by melting point analysis.

**Figure 2:**
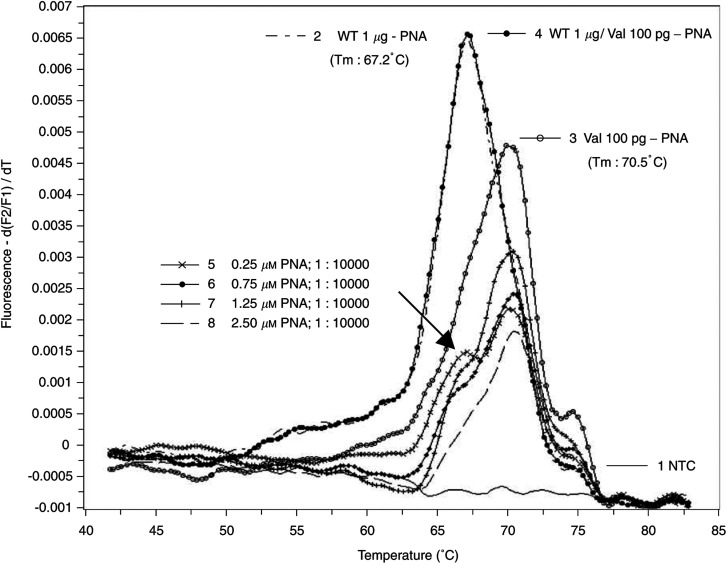
Titration of PNA concentrations for complete suppression of amplification of 1 *μ*g of wild-type DNA (WT DNA; human placenta DNA) in the presence of 100 pg of mutated DNA (10 000 : 1) extracted from cells of the colon carcinoma cell line SW 480, which bears the codon 12 mutation of the Ki-ras proto-oncogene (GGT to GTT; glycine to valine) homozygous. After rapid cycle amplification of the DNA in the presence of the valine mutation-specific hybridisation probes, the melting curves were analysed by the LightCycler software (version 3.5). Temperature transition rate was 0.3°C s^−1^. 1: non template control (NTC); 2: 1 *μ*g wild-type (WT) DNA without PNA; 3: 100 pg Val DNA without PNA; 4: 1 *μ*g WT DNA/100 pg Val DNA without PNA; 5–8: 1 *μ*g WT DNA/100 pg Val DNA each with raising concentrations of PNA as indicated. PCR experiments were performed twice.

In appropriate clinical samples the proportion of malignant or premalignant to normal cells is extremely low. As shown in Figure 2, the melting points of 100 pg of mutant DNA extracted from SW 480 colon carcinoma cells in the presence of 1 *μ*g of wild-type DNA (1 : 10 000) were not detectable (curve 2 and 4). However now, in the presence of PNA, the wild-type-specific peak disappears and the mutant allele was detectable in a 1 : 10 000 dilution (mut/wt). For complete suppression of wild-type DNA amplification, raising amounts of PNA were used. In the presence of a PNA concentration of 2.5 *μ*M in the assay (curve 8), only the mutant-specific peak was observed comparable to curve 3 of the mutation-specific peak. Lower concentrations of PNA show a weak shoulder at the wild-type-specific melting temperature (curves 5–7; arrow), indicating incomplete suppression of the wild-type DNA amplification. Under these conditions, quantification of mutant DNA is impossible. It should be noted that higher PNA concentrations lead to a decrease of the efficiency of DNA amplification and therefore lowers fluorescence intensities (data not shown).

### Determination of the sensitivity of the assay and quantification of mutated DNA

**Figure 3:**
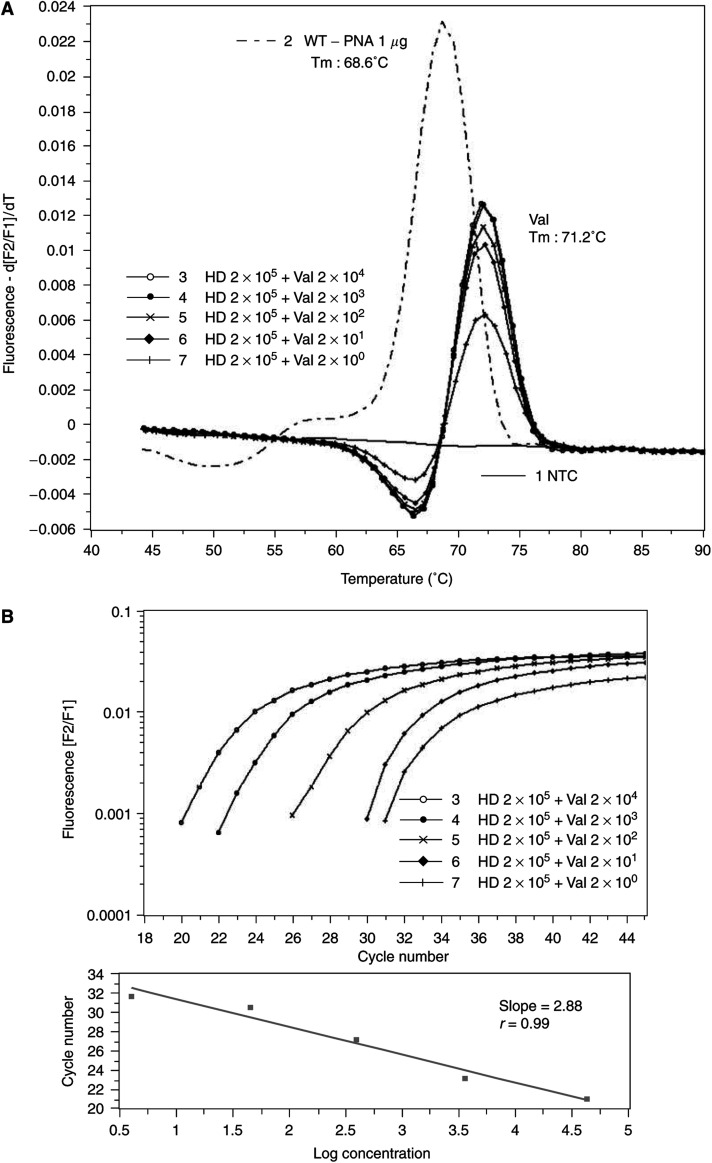
Titration of assay sensitivity. (**A**) Sensitivity of melting point analyses using mutant-specific hybridisation probes and wild-type specific PNA. Peripheral blood cells from a healthy donor (HD) and SW 480 colon carcinoma cells, which bear the valine mutation homozygous (Val), were mixed as indicated and DNA was extracted by spin column technology. After rapid cycle amplification of the DNA in the presence of the valine mutation-specific hybridisation probes, the melting curves were analysed by the LightCycler software (version 3.5). Temperature transition rate was 0.3°C s^−1^. 1: nontemplate control (NTC); 2: WT DNA without PNA; 3: 2 × 10^4^ SW480+2 × 10^5^ WT; 4: 2 × 10^3^ SW480+2 × 10^5^ WT; 5: 2 × 10^2^ SW480+2 × 10^5^ WT; 6: 2 × 10^1^ SW480+2 × 10^5^ WT; 7: 2 × 10^0^ SW480+2 × 10^5^ WT. (**B**) Quantification of Val DNA in the presence of WT DNA. Serial diluted mixtures of constant amounts of wild-type DNA and varying amounts of Val DNA from cells as indicated (see Figure 3A) were plotted against *C*_t_ values (threshold cycle). Slope, *r* value and regression line are shown. PCR experiments were performed twice.

To determine the sensitivity of our assay, serially an equivalent of 2–20 000 tumour cells of the colon carcinoma cell line SW 480, which bear the codon 12 mutation glycine (GGT) to valine (GTT) homozygous, were mixed each with 2 000 000 normal peripheral blood cells from a healthy subject. After DNA extraction, one-tenth of the eluted DNA was analysed. As seen in Figure 3A, an equivalent of two mutated cells can be detected in the presence of genomic DNA of an equivalent of 200 000 wild-type cells, which corresponds to a sensitivity of 1 to 5 : 100 000. The melting temperatures for wild-type and mutated DNA are 68.6 and 71.2°C, respectively. Additionally, the same dilution experiments were performed using enriched *BstN*I RFLP/PCR (Mora *et al*, 1998). After gel electrophoresis of the amplified products, the mutant allele (143-bp band) and wild-type allele (114-bp band) could be visualised after ethidium bromide staining. The detection limit of the mutation was 10^−3^ (data not shown). Another favourite advantage of our real-time PCR technology is the quantification of mutated DNA simultaneously, if the amplification of the wild-type DNA was completely suppressed as seen in Figure 3A. After analysing the crossing points by the LightCycler software (Figure 3B, upper panel), the regression line was linear (slope=−2.878; *r*=−0.99) in the range of 4–40 000 copy numbers of mutated DNA in the background of 400 000 copies of wild-type DNA (Figure 3B, lower panel).

### Genotyping of pancreatic tissue samples

**Figure 4:**
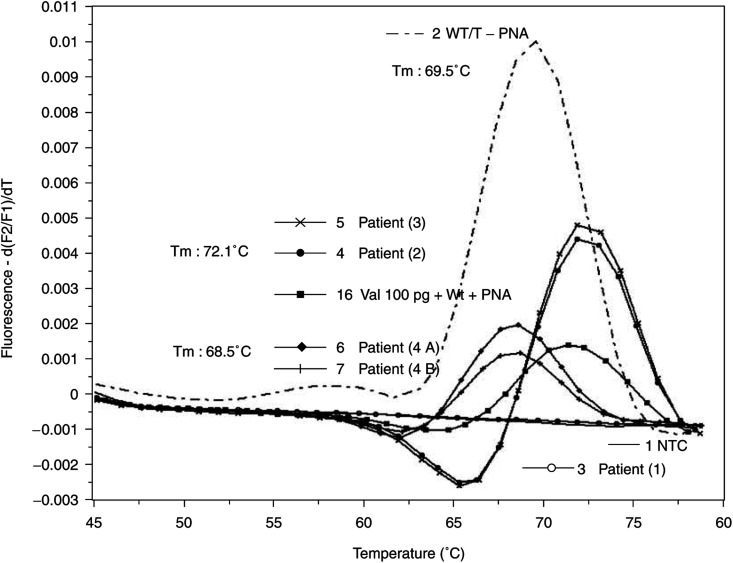
Detection of Ki-ras point mutations in tissue samples. DNA from paraffin-embedded tissue samples of four different patients with pancreatic cancer were extracted by spin column technology after overnight digestion with proteinase K. After rapid cycle amplification of the eluted DNA in the presence of the valine mutation-specific hybridisation probes and wild-type-specific PNA, the melting curves were analysed by the LightCycler software (version 3.5). Temperature transition rate was 0.3°C s^−1^ in the range of 40–95°C. PCR experiments were performed twice. Melting temperatures (*T*_m_): wild-type DNA: 69.5°C, valine DNA 72.1°C and unknown mutation 68.5°C. 1: Nontemplate control (NTC); 2: 1 *μ*g wild-type DNA (WT) without PNA; 3–7: tissue samples of patients. From patient no. 4, two different samples (4A and 4B) were analysed; 16: 100 pg Val DNA/1 *μ*g WT DNA in the presence of PNA.

Melting point analysis of amplification products was performed on five tissue samples of four patients. Patient no. 2 and 3 (Figure 4, curves 4 and 5) revealed a melting temperature of 72.1°C corresponding to a valine mutation in codon 12 of the DNA. Two different tissue samples (A and B) of patient no. 4 (Figure 4, curves 6 and 7) showed a melting point of 68.5°C representing a mutation other than valine. Further verification of this point mutation was not done. Complete suppression of amplification of wild-type DNA is demonstrated in Figure 4 (curve 16) using a ratio of mutant to wild-type DNA of 1 to 10 000. In the presence of the wild-type specific PNA, an additional tissue sample of patient no. 1 (Figure 4, curve 3) was analysed and no fluorescence signal was detected, indicating the wild-type allele. All DNAs were tested by amplification without PNA, first.

### Genotyping of plasma samples of patients with pancreatic cancer

Adequate amounts of DNA were extracted from 64 plasma samples of 10 patients (seven male, three female) with pancreatic cancer and three healthy subjects. Blood samples were obtained at least monthly over a period up to 13 months. To determine the amount of DNA per assay, real-time PCR was performed in the absence of PNA (data not shown). Owing to the low amount of extractable plasma DNA, we were able to use approximately 0.5 ng per assay. The PCR method using PNA-mediated PCR-clamping and hybridisation probes showed that the plasma samples of the three healthy control cases were negative for Ki-ras mutations as no fluorescence signals were detectable in the presence of wild-type-specific PNA. Ki-ras alterations were found in DNAs from 18 out of 64 (28%) plasma samples of four out of 10 patients with ductal adenocarcinoma of the pancreas. The mutation from glycine to valine was the predominantly detected gene alteration (15 out of 18; 83%). Other mutations were not further analysed.

Data synopsis of patients investigated is summarised in Table 1. The median was 11.5 months (5–83 months) after diagnosis. Two patients were suitable for surgical therapy. Among all patients metastases were detectable during tumour staging.

Time course analysis of the different clinical samples is shown in Table 2 in context with serum levels of the tumour marker CA 19-9, pharmacological treatments and tumour imaging.

Out of the 10 patients investigated, four patients became positive during clinical observation with respect to Ki-ras mutation (nos. 11, 34, 39, 43). In all these cases, the mutated DNA was steadily detected until death of the patients in association with tumour progression and raising CA 19-9 serum levels (>10^4^ U ml^−1^). The altered Ki-ras gene was not detected in patient no. 40, while CA 19-9 level was slightly elevated.

Three patients (nos. 13, 16, 38) show raising tumour marker values along with progressive disease, but no mutation was detectable in plasma samples. Patients nos. 29 and 37 showed progressive disease, while CA 19-9 values were low and no Ki-ras gene alterations were found.

## DISCUSSION

Our aim was to establish an assay for the detection of the hot spot mutation in codon 12 of the Ki-ras gene in plasma samples using PNA-mediated PCR-clamping and mutant-specific hybridisation probes. In contrast to Thiede *et al* (1996), where in case of mutant DNA the PCR primer outcompete the wild-type specific PNA, we used wild-type PNA (17-mer) and mutant-specific fluorescent-labelled hybridisation probes. Owing to the higher thermal instability of mutant DNA and wild-type-specific PNA hybrids, the detected fluorescence signal corresponds to the amplified mutant DNA and can be analysed by subsequent melting curve analysis.

Ki-ras mutations were analysed in a multitude of clinical specimens like fine-needle aspirates, stool, pancreatic and duodenal juice, blood cells, serum and plasma (Minamoto *et al*, 2000) with high specificity (up to 100%) and a wide spectrum of sensitivities in the range of 27–100% (Mulcahy *et al*, 1998; Castells *et al*, 1999; Li *et al*, 2004). Owing to the retroperitoneal position of the pancreas, tissue samples are difficult to obtain. Therefore, analysis of clinical samples easy and noninvasive to obtain like plasma would make molecular diagnosis easier, especially in earlier stages of pancreatic cancer and follow-up investigations. The origin of mutated plasma DNA of patients with malignant neoplasm is uncertain but due to the same gene alterations as the primary tumour it is likely that lysed tumour cells are the origin of naked DNA (Castells *et al*, 1999). The analysis of tumour cells in peripheral blood is difficult because of the rareness of neoplastic cells. Furthermore, large amounts of nonmutated leucocytes mask the vestiges of mutated cells.

We restricted our point mutation analysis to one of the preferred glycine (GGT) to valine (GTT) variant as described in literature (Anker *et al*, 1997), but our method allows the detection of the other gene alterations at codon 12 (aspartic acid, arginine and cysteine), too. The applicability of this method, first of all the correlation of detected gene mutations with clinicopathological findings, was evaluated by subsequent time course analysis of circulating non-cell-associated DNA in peripheral blood of patients with pancreatic cancer. The occurrence of Ki-ras gene mutations in primary pancreatic tumours is well known from literature (Tada *et al*, 1993; Sorenson *et al*, 1994; Mulcahy *et al*, 1998). We also could detect Ki-ras gene mutations in selected tissue samples of the pancreas using our assay.

The sensitivity of our assay revealed a value of 0.001% being the highest compared with other methods for genotyping like ASA-PCR and RFLP-PCR reaching sensitivities in the range of 1.0–0.001% (Jacobson and Mills, 1994; Rhodes *et al*, 1997). Furthermore, the rapid cycle PCR enables quantification of the mutant DNA simultaneously if the fluorescence signal of the wild-type DNA is completely suppressed by binding of the PNA.

Quantification of mutant-type Ki-ras gene in plasma samples is inappropriate due to the low amount of DNA and the absence of cellular equivalents which allows the quantification of single copy genes as a reference. This method allows a very fast detection of Ki-ras point mutations (∼1 h) after DNA preparation from clinical samples. As no post-PCR handling is necessary, the possibility of contamination is minimised, which contributes to reduce false positive results.

While all previous studies only analysed one sample per individual after diagnosis of pancreatic cancer, we present a preliminary study of time course analysis of Ki-ras gene alterations. Thus, occurrence of mutations in samples analysed in our study can hardly be compared with incidences found by other investigators (Mulcahy *et al*, 1998; Castells *et al*, 1999; Dianxu *et al*, 2002; Maire *et al*, 2002). The percentages of plasma samples with mutated Ki-ras gene found to date in the existing studies differ in a wide range of 27–81%, which might reflect collection of samples at different tumour stages and various sensitivities of the assays for detection of Ki-ras point mutations. Dianxu *et al* (2002) tested 37 of 41 patients (90.2%) with pancreatic cancer positive when plasma Ki-ras mutation analysis was combined with elevated CA 19-9 serum levels (>37 Units ml^−1^). In our study, we detected Ki-ras mutant alleles only in four out of 10 patients with high CA 19-9 levels. These differences might be due to different sensitivities of the detection methods, even though the sensitivity of our method was the highest compared to the others. In general, more clinical samples of patients with pancreatic cancer, chronic pancreatitis and healthy individuals have to be analysed for determination of sensitivity, specificity, negative and positive predictive values of the assay presented in this study. Owing to the limited number of patients analysed, a correlation of the detectable Ki-ras mutations with clinicopathological findings and pharmacological treatments is certainly prematurely, but we can demonstrate the potential of the rapid cycle PCR in the presence of wild-type PNA and mutation-specific hybridisation probes for detection of point mutations.

We could identify Ki-ras-mutated alleles by this rapid real-time PCR at late stages of carcinogenesis very well and may contribute to therapeutic regimes and clinical practice.

## Figures and Tables

**Table 1:** Patients and incidence of Ki-ras mutations in plasma DNA

**No.**	**Sex**	**Age (years)**	**Tumour site**	**Tumour stage**	**Histological type**	**Surgery**	**Survival**	**WT**	**Mut**
11	M	67	NA	IVb	DAC	No	9	2	5
13	M	81	Head	Metastases	DAC	No	6	5	0
16	M	70	Head	IVb	NA	No	35	10	0
29	M	51	Head	Metastases	DAC (moderate)	No	5	3	0
34	M	50	Head	Metastases	DAC (poorly)	No	8	3	3
37	F	69	Head	T2, N1, M1	NA	No	23	9	0
38	F	57	Head	Metastases	DAC (moderate)	No	6	4	0
39	M	59	Head	Metastases	DAC (moderate)	Yes	83	1	1
40	F	71	Head	T3, N1, M1	DAC (moderate/poorly)	Yes	43	9	0
43	M	52	Head	Metastases	DAC (moderate)	No	14	0	9

DAC=ductal adenocarcinoma; NA=data not available; Survival=survival in months after diagnosis; WT=wild-type (number of samples); Mut=mutation (number of samples).

**Table 2:** Association with clinicopathological findings

	**Months after first sample**													
**Patient**	**0**	**1**	**2**	**3**	**4**	**5**	**6**	**7**	**8**	**9**	**10**	**11**	**12**	**13**
No. 11FS 3Ex 10	Gly	Gly	**Val**	*NA*	**Val**	**Val**	**Val**	**Val**	←Ki-ras status in plasma					
	2654	2706	5288	>10^4^	8077	NA	>10^4^		←CA 19-9 level in serum (U ml^−1^)					
	GFF	GFF	Tax	Tax	Tax	Tax	Tax	Tax	←(Chemo-) therapy					
	NC		PD						←Imaging					
No. 13FS 0Ex 7	Gly	NA	Gly	*NA*	Gly	Gly	Gly							
	6879	NA	698	654	2254	>10^4^	>10^4^							
	Gem	Gem	Gem	Gem	Gem	Gem	OFF							
				PR		PD								
No. 16FS 22Ex 35	Gly	*NA*	Gly	*NA*	Gly	*NA*	Gly	Gly	Gly	Gly	Gly	*NA*	Gly	Gly
	109	62	43	NA	45	90	NA	191	340	426	1948	NA	4216	NA
	OFF	OFF	OFF	OFF	OFF	OFF	OFF	OFF	OFF	OFF	OFF	OFF	Gem	Gem
				NC				NC					PD	
No. 29FS 2Ex 5	Gly	Gly	Gly	*NA*										
	1	NA	9	32										
	Gem	OFF		OFF										
	PD			PD										
No. 34FS 1Ex 8	Gly	*NA*	Gly	Gly	**Mut**	**Mut**	**Mut**							
	5916	2291	4169	>10^4^	>10^4^	>10^5^	NA							
	Gem	Gem	Gem	Gem	Gem	OFF	OFF							
			PR		PD									
No. 37FS 11Ex 23	Gly	NA	Gly	NA	Gly	Gly	Gly	Gly	Gly	Gly	Gly			
	7	NA	13	NA	28	37	NA	41	50	52	56			
	Tax	Tax	Tax	Tax	Tax	Tax	Tax	Tax	Tax	Tax	Tax			

No. 38FS 1Ex 5	Gly	Gly	Gly	Gly										
	>10^6^	>10^6^	NA	>10^6^										
	Gem	Gem	OFF	OFF										
			PD											
No. 39FS 82Ex 83	Gly	**Val**												
	>10^4^	>10^4^												
	Gem	Gem												
	PD													
No. 40FS 31Ex 43	Gly	*NA*	NA	Gly	*NA*	Gly	Gly	Gly	Gly	Gly	Gly	*NA*	Gly	
	NA	NA	309	NA	NA	464	NA	179	NA	NA	218	NA	251	
	Gem	Gem	Gem	Gem	OFF				Tax	Tax	Tax			
				PD										
No. 43FS 1Ex 14	**Val**	*NA*	*NA*	**Val**	**Val**	**Val**	**Val**	**Val**	**Val**	**Val**	**Val**	*NA*	*NA*	
	677	NA	434	1365	2116	5231	883	446	544	1506	>10^4^	>10^4^		
	GFF	GFF	GFF	GFF	GFF	Tax	Tax	Tax	Tax	Tax	Tax			
				PD					PR					


FS=time of 1st collected sample after diagnosis (in months); Ex=death in months after collection of the 1st sample; NA=no data available; Gly=glycine (wild type); Val=valine (mutation); Mut=mutation different from valine; GFF=gemcitabine+5-fluorouracil+folinic acid; Gem=gemcitabine mono; Tax=taxol; OFF=oxaliplatin+5-fluorouracil+folinic acid; NC=no change; PD=progressive disease; PR=partial regression.

## References

[bib1] Anker P, Lefort F, Vasioukhin V, Lyautey J, Lederrey C, Chen XQ, Stroun M, Mulcahy HE, Farthing MJ (1997) K-ras mutations are found in DNA extracted from the plasma of patients with colorectal cancer. Gastroenterology 112(4): 1114–1120 9097993 10.1016/s0016-5085(97)70121-5

[bib2] Behn M, Thiede C, Neubauer A, Pankow W, Schuermann M (2000) Facilitated detection of oncogene mutations from exfoliated tissue material by a PNA-mediated ‘enriched PCR’ protocol. J Pathol 190(1): 69–75 10640994 10.1002/(SICI)1096-9896(200001)190:1<69::AID-PATH503>3.0.CO;2-P

[bib3] Berndt C, Haubold K, Wenger F, Brux B, Muller J, Bendzko P, Hillebrand T, Kottgen E, Zanow J (1998) K-ras mutations in stools and tissue samples from patients with malignant and nonmalignant pancreatic diseases. Clin Chem 44(10): 2103–2107 9761241

[bib4] Castells A, Puig P, Mora J, Boadas J, Boix L, Urgell E, Sole M, Capella G, Lluis F, Fernandez-Cruz L, Navarro S, Farre A (1999) K-ras mutations in DNA extracted from the plasma of patients with pancreatic carcinoma: diagnostic utility and prognostic significance. J Clin Oncol 17(2): 578–584 10080602 10.1200/JCO.1999.17.2.578

[bib5] Dianxu F, Shengdao Z, Tianquan H, Yu J, Ruoqing L, Zurong Y, Xuezhi W (2002) A prospective study of detection of pancreatic carcinoma by combined plasma K-ras mutations and serum CA19-9 analysis. Pancreas 25(4): 336–341 12409826 10.1097/00006676-200211000-00003

[bib6] Efthimiou E, Crnogorac-Jurcevic T, Lemoine NR (2001) Pancreatic cancer genetics. Pancreatology 1(6): 571–575 12120238 10.1159/000055865

[bib43] Gansauge S, Gansauge F, Beger HG (1996) Molecular oncology in pancreatic cancer. J Mol Med 74(6): 313–320 8862512 10.1007/BF00207508

[bib7] Ghaneh P, Kawesha A, Evans JD, Neoptolemos JP (2002) Molecular prognostic markers in pancreatic cancer. J Hepatobiliary Pancreat Surg 9(1): 1–11 12021893 10.1007/s005340200000

[bib8] Goggins M, Kern SE, Offerhaus JA, Hruban RH (1999) Progress in cancer genetics: lessons from pancreatic cancer. Ann Oncol 10(Suppl 4): 4–8 10436774

[bib9] Hermanek P (1998) Pathology and biology of pancreatic ductal adenocarcinoma. Langenbecks Arch Surg 383(2): 116–120 9641883 10.1007/s004230050102

[bib10] Hilgers W, Kern SE (1999) Molecular genetic basis of pancreatic adenocarcinoma. Genes Chromosomes Cancer 26(1): 1–12 10440999

[bib12] Jacobson DR, Mills NE (1994) A highly sensitive assay for mutant ras genes and its application to the study of presentation and relapse genotypes in acute leukemia. Oncogene 9: 553–563 8290266

[bib13] Kahn SM, Jiang W, Culbertson TA, Weinstein IB, Williams GM, Tomita N, Ronai Z (1991) Rapid and sensitive nonradioactive detection of mutant K-ras genes via ‘enriched’ PCR.amplification. Oncogene 6: 1079–1083 1676837

[bib14] Lamerz R (1999) Role of tumour markers, cytogenetics. Ann Oncol 10(Suppl 4): 145–149 10436809

[bib15] Lay MJ, Wittwer CT (1997) Real-time fluorescence genotyping of factor V Leiden during rapid-cycle PCR. Clin Chem 43(12): 2262–2267 9439442

[bib16] Li D, Xie K, Wolff R, Abbruzzese JL (2004) Pancreatic cancer. Lancet 363(9414): 1049–1057 15051286 10.1016/S0140-6736(04)15841-8

[bib17] Lyon E (2001) Mutation detection using fluorescent hybridization probes and melting curve analysis. Expert Rev Mol Diagn 1(1): 92–101 11901805 10.1586/14737159.1.1.92

[bib18] Maire F, Micard S, Hammel P, Voitot H, Levy P, Cugnenc PH, Ruszniewski P, Puig PL (2002) Differential diagnosis between chronic pancreatitis and pancreatic cancer: value of the detection of KRAS2 mutations in circulating DNA. Br J Cancer 87(5): 551–554 12189555 10.1038/sj.bjc.6600475 PMC2376157

[bib19] Malats N (2001) Gene–environment interactions in pancreatic cancer. Pancreatology 1(5): 472–476 12120227 10.1159/000055850

[bib20] Minamoto T, Mai M, Ronai Z (2000) K-ras mutation: early detection in molecular diagnosis and risk assessment of colorectal, pancreas, and lung cancers – a review. Cancer Detect Prev 24(1): 1–12 10757118

[bib21] Mizumoto K, Tanaka M (2002) Genetic diagnosis of pancreatic cancer. J Hepatobiliary Pancreat Surg 9(1): 39–44 12021896 10.1007/s005340200003

[bib22] Mora J, Puig P, Boadas J, Urgell E, Montserrat E, Lerma E, González-Sastre F, Lluís F, Farré A, Capellá G (1998) K-ras gene mutations in the diagnosis of fine-needle aspirates of pancreatic masses: prospective study using two techniques with different detection limits. Clinical Chem 44: 2243–2248 9799749

[bib24] Mulcahy HE, Lyautey J, Lederrey C, qi Chen X, Anker P, Alstead EM, Ballinger A, Farthing MJ, Stroun M (1998) A prospective study of K-ras mutations in the plasma of pancreatic cancer patients. Clin Cancer Res 4(2): 271–275 9516910

[bib25] Olson MF, Marais R (2000) Ras protein signalling. Semin Immunol 12(1): 63–73 10723799 10.1006/smim.2000.0208

[bib26] Ørum H, Nielsen PE, Egholm M, Berg RH, Buchardt O, Stanly C (1993) Single base pair mutation analysis by PNA directed PCR clamping. NAR 21: 5332–5336 8265345 10.1093/nar/21.23.5332 PMC310567

[bib27] Ratilainen T, Holmen A, Tuite E, Haaima G, Christensen L, Nielsen PE, Norden B (1998) Hybridization of petide nucleic acid. Biochemistry 37: 12331–12342 9724547 10.1021/bi9808722

[bib28] Rhodes CH, Honsinger C, Porter DM, Sorenson GD (1997) Analysis of the allele-specific PCR method for the detection of neoplastic disease. Diagn Mol Pathol 6: 49–57 9028737 10.1097/00019606-199702000-00008

[bib29] Sakorafas GH, Tsiotou AG, Tsiotos GG (2000) Molecular biology of pancreatic cancer; oncogenes, tumour suppressor genes, growth factors, and their receptors from a clinical perspective. Cancer Treat Rev 26(1): 29–52 10660490 10.1053/ctrv.1999.0144

[bib30] Shi X, Friess H, Kleeff J, Ozawa F, Buchler MW (2001) Pancreatic cancer: factors regulating tumor development, maintenance and metastasis. Pancreatology 1(5): 517–524 12120231 10.1159/000055854

[bib31] Simon B, Printz H (2001) Epidemiological trends in pancreatic neoplasias. Dig Dis 19(1): 6–14 11385246 10.1159/000050648

[bib32] Sirivatanauksorn V, Sirivatanauksorn Y, Lemoine NR (1998) Molecular pattern of ductal pancreatic cancer. Langenbecks Arch Surg 383(2): 105–115 9641882 10.1007/s004230050101

[bib44] Sorenson GD, Pribish DM, Valone FH, Memoli VA, Bzik DJ, Yao SL (1994) Soluble normal and mutated DNA sequences from single-copy genesin human blood. Cancer Epidemiol Biomarkers Prev 3(1): 67–71 8118388

[bib33] Sotlar K, Escribano L, Landt O, Mohrle S, Herrero S, Torrelo A, Lass U, Horny HP, Bultmann B (2003) One-step detection of c-kit point mutations using peptide nucleic acid-mediated polymerase chain reaction clamping and hybridization probes. Am J Pathol 162(3): 737–746 12598308 10.1016/S0002-9440(10)63870-9 PMC1868096

[bib35] Sternheim ET, Voigt J, Kaspar W, Dippold WG (2000) Pancreatic carcinoma. Internist (Berl) 41(9): 848–854, 856–859 11006872 10.1007/s001080050637

[bib45] Tada M, Omata M, Kawai S, Saisho H, Ohto M, Saiki RK, Sninsky JJ (1993) Detection of ras gene mutations in pancreatic juice and peripheral blood of patients with pancreatic adenocarcinoma. Cancer Res 53(11): 2472–2474 8495407

[bib36] Takeda S, Ichii S, Nakamura Y (1993) Detection of K-ras mutation in sputum by mutant-allele-specific amplification (MASA). Hum Mutat 2(2): 112–117 8318987 10.1002/humu.1380020209

[bib38] Thiede C, Bayerdorffer E, Blasczyk R, Wittig B, Neubauer A (1996) Simple and sensitive detection of mutations in the ras proto-oncogenes using PNA-mediated PCR clamping. Nucleic Acids Res 24(5): 983–984 8600471 10.1093/nar/24.5.983 PMC145732

[bib39] Weber JL (1990) Human DNA polymorphisms and methods of analysis. Curr Opin Biotechnol 1: 166–171 1367853 10.1016/0958-1669(90)90026-h

[bib40] Wenger FA, Zieren J, Peter FJ, Jacobi CA, Muller JM (1999) K-ras mutations in tissue and stool samples from patients with pancreatic cancer and chronic pancreatitis. Langenbecks Arch Surg 384(2): 181–186 10328172 10.1007/s004230050189

[bib41] Wong T, Howes N, Threadgold J, Smart HL, Lombard MG, Gilmore I, Sutton R, Greenhalf W, Ellis I, Neoptolemos JP (2001) Molecular diagnosis of early pancreatic ductal adenocarcinoma in high-risk patients. Pancreatology 1(5): 486–509 12120229 10.1159/000055852

